# Designing Electronic Data Capture Systems for Sustainability in Low-Resource Settings: Viewpoint With Lessons Learned From Ethiopia and Myanmar

**DOI:** 10.2196/47703

**Published:** 2024-02-12

**Authors:** Natalie Benda, Kylie Dougherty, Abebe Gebremariam Gobezayehu, John N Cranmer, Sakie Zawtha, Katerina Andreadis, Heran Biza, Ruth Masterson Creber

**Affiliations:** 1 School of Nursing Columbia University New York, NY United States; 2 Emory-Ethiopia Partnership Bahir Dar Ethiopia; 3 Bahir Dar University Bahir Dar Ethiopia; 4 Nell Hodgson Woodruff School of Nursing Emory University Atlanta, GA United States; 5 Health and Hope Myanmar Lailenpi Myanmar; 6 New York University Grossman School of Medicine New York, NY United States

**Keywords:** low and middle income countries, LMIC, electronic data capture, population health surveillance, sociotechnical system, data infrastructure, electronic data system, health care system, technology, information system, health program development, intervention

## Abstract

Electronic data capture (EDC) is a crucial component in the design, evaluation, and sustainment of population health interventions. Low-resource settings, however, present unique challenges for developing a robust EDC system due to limited financial capital, differences in technological infrastructure, and insufficient involvement of those who understand the local context. Current literature focuses on the evaluation of health interventions using EDC but does not provide an in-depth description of the systems used or how they are developed. In this viewpoint, we present case descriptions from 2 low- and middle-income countries: Ethiopia and Myanmar. We address a gap in evidence by describing each EDC system in detail and discussing the pros and cons of different approaches. We then present common lessons learned from the 2 case descriptions as recommendations for considerations in developing and implementing EDC in low-resource settings, using a sociotechnical framework for studying health information technology in complex adaptive health care systems. Our recommendations highlight the importance of selecting hardware compatible with local infrastructure, using flexible software systems that facilitate communication across different languages and levels of literacy, and conducting iterative, participatory design with individuals with deep knowledge of local clinical and cultural norms.

## Introduction

Information systems supporting electronic data capture (EDC) for research and clinical operations are crucial to the development, evaluation, and sustainability of population health programs, facilitating health improvements supported by data. In some low- and middle-income countries (LMIC), EDCs can trend health outcomes and inform decision-making, thereby improving the efficiency and effectiveness of health services [1,2]. EDC can support health intervention and policy development along with determining the allocation of critical resources [3]. With the expansion of internet services globally, using EDC systems is a cost-effective way to increase the amount and quality of data that can be used in LMIC [4,5].

However, EDC will only be useful in promoting positive health outcomes when the system matches the required users, tasks, and environment [6]. Implementing EDC systems to monitor health indicators is a complex process. It requires that EDC systems be developed to (1) suit the needs of the local context, (2) support long-term sustainability, and (3) accommodate routine adaptations [7-9]. Barriers to successful development include limited financial capital for implementation, low utilization of local expertise, and narrow conceptualizing of EDC technologies while ignoring relevant sociotechnical factors that should drive EDC decisions [10].

Published information on health interventions in LMIC typically focuses on the results of the interventions themselves rather than the EDC used for population health surveillance. Therefore, guidance for developing EDC systems is limited [10]. One qualitative study, for example, evaluating the implementation of an EDC, described the lack of trained information system specialists, inconsistent definitions of variables, insufficient data validation, managers not valuing the data, and communication issues as the core challenges to EDC implementation [10]. One systematic review found 132 published studies that used health information systems for data collection in LMIC [3]. The study reported on data analysis methods and data quality, noting that data completeness was a commonly cited issue with EDCs and exclusion, followed by imputation, was the most common means for handling missing data [3]. These publications highlight a common pattern among studies regarding EDC in LMIC, that is, if they go beyond simply describing the impacts of the interventions, the primary focus involves data quality and completeness. Although quality and completeness are important elements to evaluate with EDC, there remains a gap in describing the nature of the EDC systems and how their components were assembled and implemented.

Due to this gap in evidence, the objective of this viewpoint is to provide practical recommendations for designing EDC systems for research and health operations to sustain data-informed population health interventions globally. We describe 2 exemplar cases in Ethiopia and Western Myanmar/Eastern India to illustrate how robust EDC systems may be designed to support population health surveillance, specifically during humanitarian crises (ie, civil unrest and the COVID-19 pandemic). The research activities related to these cases have been described in other publications [11,12]. In this viewpoint, we describe case descriptions using a sociotechnical conceptual model to determine barriers and facilitators of successful development and use of EDC. We used this model because it provides a comprehensive framework that can be used to guide and evaluate EDC design and implementation [13].

## Context and Project Overviews

### Ethiopia Case Description–The Saving Little Lives Initiative

As of 2019, Ethiopia had high ratios of neonatal mortality (33 per 1000 live births) and infant mortality (47 per 1000 live births) [14]. Globally, Ethiopia has the fourth-highest number of newborn deaths [15]. Further, the Sub-Saharan Africa region has the highest neonatal mortality ratio in the world [15].

Ethiopia-specific causes of neonatal death include respiratory distress (45%), infection (30%), and birth asphyxia (13%) [14]. To improve newborn and infant health, the Emory-Ethiopia Partnership began work in Amhara in 2011. This case description focuses on one of Emory-Ethiopia’s efforts in the region—Saving Little Lives (SLL). SLL is a flagship health program of the Ethiopian government that spans 290 targeted hospitals across 4 regions. SLL is an aggressive scale-up study designed to reduce population-level mortality by 35% through improved newborn care. SLL uses a package of targeted and synergistic interventions to promote survival. SLL consortium partners are supporting the regional health bureaus to scale up 5 key interventions, as follows: (1) respiratory support (including resuscitation for newborns with asphyxia and continuous positive airway pressure for respiratory distress), (2) kangaroo mother care (KMC), (3) sepsis management, (4) feeding support, and (5) cross-cutting quality improvement methods. The project covers a population of over 37 million individuals in 4 Ethiopian regions and 285 woredas (districts). Emory-Ethiopia is the lead partner for the Amhara region in this national SLL consortium. These SLL activities build upon the same consortium’s scale-up research on KMC conducted in the same regions [12,16].

As of September 2022, Ethiopia had nearly 500,000 confirmed cases and over 7500 deaths from COVID-19. In November 2020, following claims that a minority party, Tigray People’s Liberation Front, had attacked a federal army base, the majority party ordered a military offensive in Tigray [17]. This ongoing conflict has led to the forced displacement of over 2 million civilians and a food crisis affecting at least 9.4 million northern Ethiopians—primarily in the Tigray region [18]. Furthermore, the crisis has led to an increase in gender-based violence and a decrease in access to proper health services for women and children [18]. Research associated with this case was approved by institutional review boards (IRBs) in the United States and Ethiopia.

### Eastern India and Western Myanmar Description–The Mobile Health (mHealth) and Mobile Ultrasound for Mothers (mMUM)

Western Myanmar has one of the highest maternal mortality ratios in Asia, ranking 148 out of 187 on the 2019 Human Development Index [19,20]. Among women of reproductive age, maternal deaths account for 1 in every 10 deaths, with 75% occurring during delivery or immediately after birth [19].

On February 1, 2021, the Tatmadaw (Myanmar’s military) seized control of the government in a coup d’état. Health care professionals across Myanmar have participated in the Civil Disobedience Movement in response to the overthrow of a democratically elected government. As such, many women in Myanmar have even less access to obstetric care and births attended by health care professionals. Broader safety concerns following Myanmar’s military coup have also led the Mobile Health (mHealth) and Mobile Ultrasound for Mothers (mMUM) study team to temporarily relocate efforts, working with another not-for-profit in Eastern India, where women face similar accessibility barriers.

This case description focuses on a partnership between Columbia University School of Nursing and a not-for-profit founded by local health professionals from Western Myanmar, where the team was also based. The goal of the not-for-profit is to improve the health and well-being of the rural population in poor conditions in Western Myanmar. The purpose of the mMUM study is to (1) establish an mHealth-based research data collection infrastructure and (2) implement interventions (eg, obstetric ultrasound) to improve maternal and infant health. The various aspects of research associated with this case have been approved by IRBs in the United States and India.

## Information Infrastructure Design and Adaptations

**Figure 1:**
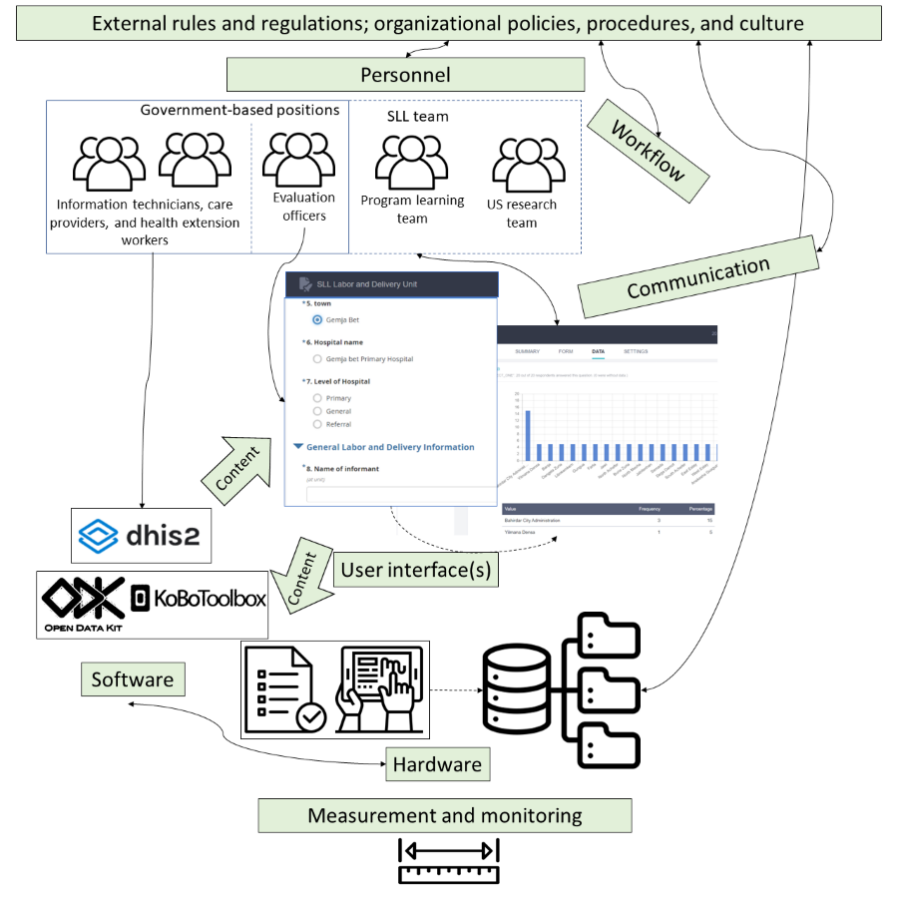
Electronic data capture infrastructure for the Saving Little Lives (SLL) initiative.

**Figure 2:**
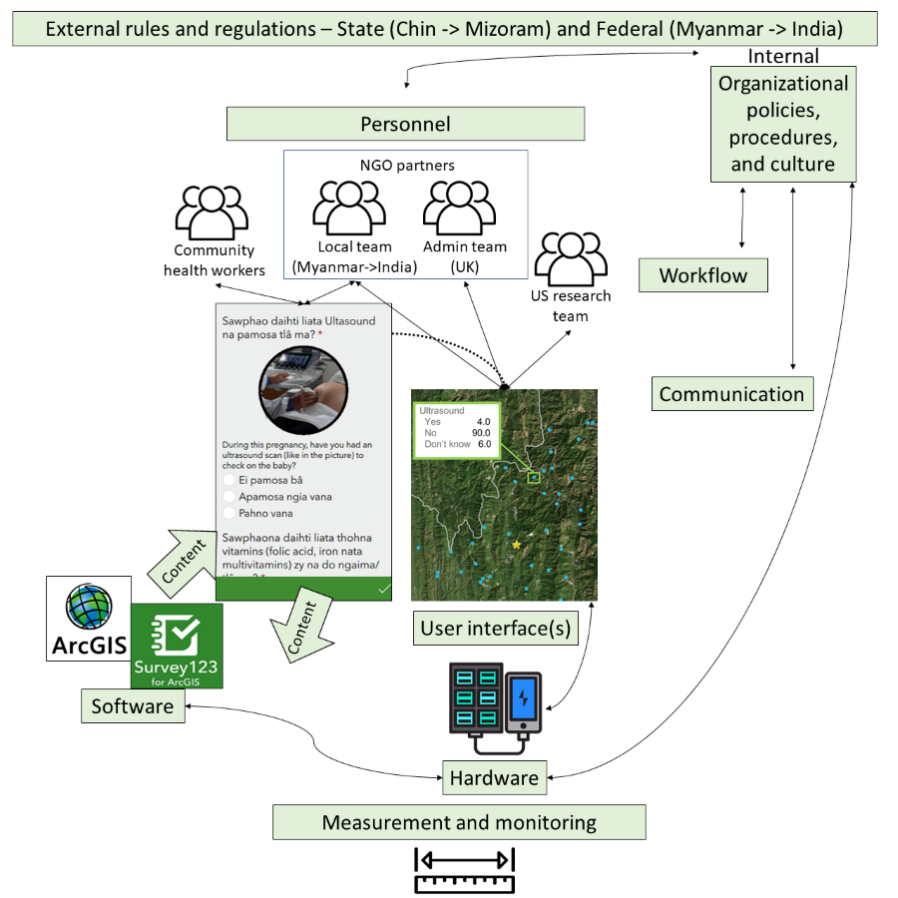
Electronic data capture infrastructure for the Mobile Health and Mobile Ultrasound for Mothers (mMUM) study. NGO: nongovernmental organization.

Sociotechnical conceptual models may provide a structure for discussing EDC considerations in LMIC—particularly those settings impacted by civil conflict. Figures 1 and 2 show the information infrastructure for each case using Sittig and Singh’s “sociotechnical model for studying health information technology in complex adaptive healthcare systems” [13]. Labeled boxes represent established components in the Sittig and Singh’s model, and the descriptions and symbols highlight how these components were operationalized in each context. Tables 1 and 2 further describe the actions taken to create and sustain these EDCs during dual humanitarian crises organized by the constructs in the Sittig and Singh’s model.

**Table 1:** Electronic data capture (EDC) design and implementation features to support population health surveillance for the Saving Little Lives (SLL) initiative [16,24,25].

Sociotechnical dimension	SLL EDC implementation activities and design features
Hardware and software computing infrastructure	Used KoBo Toolbox for easy-to-design, adaptable data collection forms. Leveraged ODK^a^ for open access sharing, allowing for the easy addition of more measures as needed to streamline data collection updates across sites. Collected data primarily via tablet and used paper to list mothers and babies discharged for further follow-up on the 29th day of life.
Clinical content	Designed content and metrics to ensure information was relevant to the local context while promoting internal and external validity (eg, incorporating clinical cascade metrics for newborns and mothers into routine SLL data collection) [16,24]. Developed programs and data collection to adhere to and be evaluated against standard quality guidelines, such as BEmONC^b^ quality or newborn cascades.
Human computer interface	Data collection forms developed with local partners and researchers based in the United States. Embedded Amharic language into data collection (eg, patient consent forms).
People	The SLL initiative funds government-based evaluation officer positions with responsibilities including primary data collection, data training, and ensuring facility-specific data are submitted to central database. Implementation officers support the training and implementation of SLL at the facility. Program learning officers collect qualitative information (eg, enablers and barriers) from different units and care providers.
Workflow and communication	Evaluation officers complete real-time data collection of project indicators; program learning team obtains qualitative information from families and clinicians for continuous improvement. Existing government staff at hospitals collect and enter facility-specific data into the electronic national monitoring system (ie, DHIS2^c^). Community-based health extension workers collect governmental surveillance, service provision data, and other community needs assessments using the community health information system. Community health workers record data on paper. These data are then submitted to their supervising health center, which, in turn, forwards them to the woreda (district) health office. The woreda health office aggregate these data electronically and shares them with the primary health care unit. These electronic data directly enter the national database, and the regional government can query this database for their region.
Internal and organizational policies, procedures, and culture; external rules, regulations, and pressure	Funding embedded government staff members (eg, evaluation officers) enables the synchronization of SLL data collection with government health surveillance. SLL deliberately involves university-based colleagues (eg, Emory University) so that the program may generate transferrable research findings in addition to its local public health and clinical impact. All SLL-specific data collection and procedures were approved by 3 institutional review boards from Emory University, Addis Ababa University, and Amhara Public Health Institute.
System measurement and monitoring	Leveraging DHIS2 surveillance data collected via the government’s Health Management Information System. Qualitative and quantitative evaluation via government-embedded evaluation officers who collect real-time SLL data outside the government’s DHIS2 system. Data collected by evaluation officers, program learning officers, health care providers, and health extension workers integrated by the Amhara SLL research team for comprehensive SLL program evaluation at multiple levels (eg, district, regional, or national levels).

^a^ODK: Open Data Kit.

^b^BEmONC: Basic Emergency Obstetric and Neonatal Care.

^c^DHIS2: District Health Information Software 2.

**Table 2:** Electronic data capture (EDC) design and implementation features to support population health surveillance for the Mobile Health (mHealth) and Mobile Ultrasound for Mothers (mMUM) study [25].

Sociotechnical dimension	mMUM EDC implementation activities and design features
Hardware and software computing infrastructure	Selected data collection tools (ie, ArcGIS and Survey123) that work in settings with limited internet connectivity or no internet. Purchased chargers that worked with local power sources (ie, solar, due to insufficient access to electricity). Selected software with mapping capabilities to locate, support, and track transient populations.
Clinical content	Developed data collection content with local personnel to ensure clinical and social questions were asked in a culturally sensitive manner. The content of the collected data was adapted to suit the situation in a new country with different infrastructure [25]. Added medical anthropologist to the team to provide insights into how clinical problems are perceived and communicated in the given setting.
Human computer interface	Chose a tool that also supported icon-based communication to provide context clues to users with lower literacy. Chose a tool that could provide an interface not only in multiple languages but also supported languages with non-Roman characters.
People	Used iterative participatory design process to create a culturally appropriate user interface. Worked with local stakeholders to devise a plan to motivate data collection, specifically building on the strong sense of community and crafting messaging regarding how data collection can support the community.
Workflow and communication	Local teams and community health workers handled most of the data collection due to lack of regular government surveillance data that may be used to track outcomes. Planned to complete research data collection with those who worked most closely with patients (local nongovernmental organization and community health workers) but also involved higher-level providers (eg, midwives) who would do more advanced procedures (eg, ultrasound). Pivoted to work in tandem with government-funded community health workers in India but are focusing the local team’s efforts on refugee camps, where existing health workers are struggling to have the bandwidth to provide support.
Internal and organizational policies, procedures, and culture	Had to determine explicit language and marking for ownership of data collection devices, such as phones and ultrasound machines. Added security protections to data collection devices in case they were stolen or intercepted, including encryption, dual-factor authentication, and remote wipe capabilities.
External rules, regulations, and pressure	Developed memoranda of understanding with local governments to conduct data collection and research operations. Adapted goals and data collection procedures based on local laws.
System measurement and monitoring	Developed a procedure for assessing data quality using plausibility ratings by local experts since there is no ground truth with which we can compare the data collected. We have been and will continue to collect qualitative data regarding how research data collection may impact workflow and productivity. used government-collected data in the new location [25] to assess the most pressing health issues (eg, anemia), identify areas of greatest need, and detect possible shortcomings in data collection.

### The SLL Initiative (Ethiopia)–Initial EDC System

The EDC used to evaluate SLL’s progress stems both from government health systems and SLL-dedicated staff—the program learning team and evaluation officers (Figure 1). Health Information technicians and other care providers at health facilities routinely collect government data, including information on health service utilization and clinical outcomes (eg, newborn mortality and postnatal care uptake) using the government’s District Health Information Software 2 (DHIS2) [21]. Evaluation officers are government-based staff, funded by SLL, who capture information on SLL-specific quality indicators related to clinical care (eg, uptake of KMC and provision of skin-to-skin contact). Evaluation officers visit SLL facilities and enter these data in the KoBo Toolbox (described below) with measures aggregated in a centralized data warehouse [22]. As partners with the Ethiopian Ministry of Health, SLL partners have access to routine government data for their region from the DHIS2 system. The SLL program staff access both these routine DHIS2 data and SLL-specific program data. These multisource data facilitate the optimization of interventions using both internal and external data sources. Refinement and utilization of EDC procedures have also directly informed core content areas in the national Basic Emergency Obstetric and Neonatal Care guidelines [23].

The data collection platform for SLL has been developed using KoBo Toolbox and an Open Data Kit (ODK) tool. The KoBo Toolbox allows for open-source sharing of input forms and enables users to design data collection forms. Forms designed in the KoBo Toolbox can be imported into ODK, which provides a graphical user interface for simple data collection based on the forms input from KoBo.

Evaluation officers collect SLL-specific data using tablets with the ODK interface and forms and submit the data to the KoBo server. Each of them has a power bank for charging and airtime to upload data into the web-based server. All the collected data are fed into a centralized database to support collaboration and evaluation at multiple levels (ie, district, regional, and national levels as well as for scientific analysis).

Evaluation officers also use a collateral data collection strategy. They have printed tracking sheets that include information such as the name of the mother, her address, her phone number, or family members’ phone numbers, allowing them to call and gather information on the status of the mother and newborn.

Table 1 describes the features of the SLL EDC infrastructure.

### The mMUM Study (Western Myanmar/Eastern India)–Initial EDC System

To design the mMUM study’s EDC system, we selected the ArcGIS (Esri) mapping software coupled with a survey platform (Survey123) [26] allowing community health workers and local nongovernmental organization team members to easily collect data in areas with low internet connectivity or no internet (Figure 2). ArcGIS uses cloud-based data storage. This helped support the local data collection and analysis from team members not working at the local sites. However, the location of the entity providing the cloud storage (ArcGIS in this case, located in the United States) came into question during the local IRB approval process. Cloud data storage approval was eventually granted, as only coded data and not personal health identifiers would be saved. The data collection forms allow for the inclusion of different languages and pictures to facilitate form comprehension. The survey software supports mobile apps for field-based data capture. The mapping software facilitates aggregation and embeds data into high-quality geographical information systems for tracking transient populations (eg, displaced person groups).

Table 2 provides additional details regarding the EDC features for the mMUM study.

The mMUM study required further adaptations following the move from Myanmar to India, facilitated by the design of the EDC system in place, as highlighted based on relevant sociotechnical dimensions described below.

#### Clinical Content

Our team has worked with local organizations from Myanmar and those native to India to update the content of the questions asked to patients in the data collection instruments. For example, new questions related to refugee status have been added, and questions pertaining to access to health facilities have been omitted, considering the stability and well-established nature of health facilities in India.

#### Human-Computer Interface

Our selection of a tool that could be translated into multiple languages has been helpful in relocation efforts, as the materials had to be translated into an additional language to support the Indian health workers.

#### Workflow and Communication

Based on discussions with Indian government officials, local team efforts leverage the medical expertise of those who are also Burmese refugees. This enables them to collect data and provide interventions to pregnant refugees, a population the Indian health system has found challenging to support due to the large and initially unexpected influx of patients in need. The Burmese aid workers also have the linguistic and cultural competency to support the unique needs of the refugee patients.

#### External Rules, Regulations, and Pressure

Our efforts have involved gaining memoranda of understanding with state-level government health officials in both Myanmar (initially) and India (adaptation). We have also had to adapt our interventions slightly, as in Myanmar we had planned to use portable ultrasound to serve rural areas. Portable ultrasound, however, is illegal in India due to issues with unregistered devices being used for gender selection (ie, female feticide). Therefore, our team has shifted focus to ensuring women are aware of where they can access legal ultrasounds and have transportation. We also plan to implement additional interventions that leverage the information we are collecting to better support the needs of pregnant refugees.

#### System Measurement and Monitoring

Myanmar did not have enough frequent public health surveillance data to help inform and pinpoint our team’s efforts. However, the population health surveillance in India is much more robust (and publicly available), allowing us to use this information to determine which districts are most in need.

## Discussion of Lessons Learned

### Overview

The 2 cases discussed involve curating EDC, considering various interacting sociotechnical dimensions to design EDC infrastructure for population health surveillance. This infrastructure is aimed at supporting research and clinical activities in 2 LMIC. Robust system design allowed our team to continue operations and make adaptations in varied capacities through close collaboration with local partners. This provided crucial data-informed health information relevant to vulnerable newborns, women, and families in these 2 LMIC despite ongoing humanitarian crises. The ability of both the mMUM and SLL projects to continue despite the dual humanitarian-COVID-19 crises was facilitated by the design of sustainable EDC infrastructure, which provided a foundation for each project’s interventions. There are transferrable lessons on optimizing EDC design for research and public health or clinical practice in LMIC, emerging from these 2 cases.

Despite differences in context, common lessons may be drawn from the 2 exemplar cases. Table 3 provides recommendations synthesized from our collective experience to guide EDC development and implementation in LMIC in future cases, organized using the dimensions of Sittig and Singh’s framework [13]. Both cases had strong foundations based on technical dimensions (eg, hardware, software, and human-computer interfaces) that supported necessary social, cultural, and organizational implementation. Multiple recommendations stress the importance of directly involving those with a deep understanding of clinical context, people (end users), and cultural norms in the EDC development and implementation process. These recommendations align with the current literature, underscoring the importance of leveraging an iterative design approach when developing EDC and involving local end users and experts [27-29]. Others have also described the benefits of KoBo’s ODK and its ability to support EDC in settings with low internet connectivity [28]. However, previous studies typically describe EDC in the context of a single, specific system implementation without describing general important considerations, as is done in this viewpoint. Both cases involved engaging with government officials and data, although the SLL team has greater integration with the Ethiopian government. The mMUM team has gained approvals to work with government-funded community health workers in certain districts and has been able to use government-collected outcomes for maternal and child health in India to guide EDC development and variable selection. However, direct integration, as accomplished by the SLL team, is more challenging due to the displacement of some team members from Myanmar.

**Table 3:** Lessons learned for electronic data capture (EDC) development from the described case studies.

Sociotechnical dimension	Recommendation for EDC Development
Hardware and software computing infrastructure	Consider data collection tools that function in scenarios with low or no internet connectivity. Plan for necessary device charging resources, particularly for settings with limited electricity or power outages. Leverage existing data infrastructure (eg, DHIS2^a^) and open-source tools (eg, KoBo Toolbox).
Clinical content	Ensure there is an understanding of important perceptions of the clinical conditions evaluated and how clinical concepts may be best translated into local languages.
Human-computer interface	Conduct iterative participatory design with end users who deeply understand the context to ensure system usability and feasibility. Make sure the platforms support different linguistic needs. Consider whether symbolic communication is supported for those with lower literacy levels.
People	Ensure some of those involved in system development are familiar with and work directly in the local settings (similar to the “clinical content” dimension).
Workflow and communication	Engage in regular, continued communication among partners (both local and abroad, as applicable) throughout implementation.
Internal and organizational policies, procedures, and culture	Involve those who can support ensuring buy-in from necessary local stakeholders, such as community health workers and patients (similar to the “clinical content” and “people” dimensions). Include those with relevant cultural expertise (eg, in medical anthropology) who can support the development of culturally congruent and sensitive data collection systems.
External rules, regulations, and pressure	Leverage local and governmental partnerships to understand and work within the target regions’ regulations.
System measurement and monitoring	Define data security and storage procedures in advance. Use governmental data for measurement (in the SLL^b^ case) or as a gold standard comparison point (in the mMUM^c^ case) when available.

^a^DHIS2: District Health Information Software 2.

^b^SSL: Saving Little Lives.

^c^mMUM: mHealth and Mobile Ultrasound for Mothers.

Compared to SLL, the mMUM study required more extensive adaptations to continue the project. This may be due to several factors, including the project being in earlier phases and less well-established than SLL as well as the need to relocate to a different country altogether due to a national government coup. Myanmar also represents a particularly challenging context from the perspective of developing EDC systems, as the country was essentially closed to external global influence until 2014. Consequently, other global countries with more technology-related partner engagement may have further developed EDC systems at baseline compared to Myanmar.

### Conclusions

This viewpoint addresses a gap in the literature by providing concrete recommendations for the development and refinement of EDC in LMIC, supported by case descriptions from 2 projects. In each case, the EDC infrastructure developed allowed the teams to continue clinical and research-based operations through humanitarian crises, including the COVID-19 pandemic and civil unrest. Common themes from each case have been presented as a series of recommendations for future EDC development and implementation in LMIC. Local system descriptions and recommendations leverage Sittig and Singh’s “sociotechnical model for studying health information technology in complex adaptive healthcare systems” [13], suggesting this model may be helpful for LMIC settings in addition to its previous use in high-income countries. Key elements of our recommendations include selecting hardware that accommodates local infrastructure, selecting systems that allow for communication across language and literacy levels, conducting iterative design with those with deep local and contextual knowledge throughout the design process, as well as gaining approval and buy-in from governmental entities.

## Abbreviations

DHIS: District Health Information Software
EDC: electronic data capture
IRB: institutional review board
KMC: kangaroo mother care
LMIC: low- and middle-income countries
mHealth: mobile health
mMUM: mHealth and Mobile Ultrasound for Mothers
ODK: Open Data Kit
SSL: Saving Little Lives
